# Longitudinal changes in home confinement and mental health implications: a 17-month follow-up study in England during the COVID-19 pandemic

**DOI:** 10.1017/S0033291722000605

**Published:** 2023-07

**Authors:** Feifei Bu, Andrew Steptoe, Daisy Fancourt

**Affiliations:** Department of Behavioural Science and Health, Institute of Epidemiology & Health Care, University College London, London, UK

**Keywords:** Anxiety, COVID-19, depression, growth mixture model, isolation, panel data

## Abstract

**Background:**

The coronavirus disease 2019 (COVID-19) pandemic has brought about significant behavioural changes, one of which is increased time spent at home. This could have important public health implications. This study aimed to explore longitudinal patterns of ‘home confinement’ (defined as not leaving the house/garden) during the COVID-19 pandemic, and the associated predictors and mental health outcomes.

**Methods:**

Data were from the UCL COVID-19 Social Study. The analytical sample consisted of 25 390 adults in England who were followed up for 17 months (March 2020–July 2021). Data were analysed using growth mixture models.

**Results:**

Our analyses identified three classes of growth trajectories, including one class showing a high level of persistent home confinement (the home-confined, 24.8%), one changing class with clear alignment with national containment measures (the adaptive, 32.0%), and one class with a persistently low level of confinement (the unconfined, 43.1%). A range of factors were associated with the class membership of home-confinement trajectories, such as age, gender, income, employment status, social relationships and health. The home-confined class had the highest number of depressive (diff = 1.34–1.68, *p* < 0.001) and anxiety symptoms (diff = 0.84–1.05, *p* < 0.001) at the end of the follow-up than the other two classes.

**Conclusions:**

There was substantial heterogeneity in longitudinal patterns of home confinement during the COVID-19 pandemic. People with a persistent high level of confinement had the worst mental health outcomes, calling for special attention in mental health action plans, in particular targeted interventions for at-risk groups.

## Introduction

Since December 2019, the world has been devastated by coronavirus disease 2019 (COVID-19). To control the spread of the disease, in addition to actions such as hand hygiene, face covering and social distancing, lockdowns and ‘stay-at-home’ orders were carried out in many countries (Hale et al., 2021). During lockdowns, people were typically asked to shelter at home, except in circumstances of necessity, with most workplaces, schools and non-essential businesses being closed for extended periods (1–3 months). These specific lockdowns suggest on the surface that individuals followed similar patterns of how much time their spent in their homes. However, individual behaviours varied substantially due to, for example, clinical vulnerabilities or non-compliance (Fancourt, Bu, Mak, & Steptoe, 2020; Smith et al., 2020). Although during lockdowns people were typically allowed to leave their homes for essential reasons including exercise, reports have suggested that many people did not leave their homes at all each day, thereby engaging in ‘home confinement’. Further, wide individual variations are expected after lockdowns, with restrictions being eased or as the number of COVID-19 cases changed. However, to date, there has been no research exploring what proportion of people engaged in home confinement and how their patterns of confinement changed across the pandemic, during and after national lockdowns, taking into account potentially heterogeneity in longitudinal changes in home confinement.

‘Home confinement’ is an important behaviour to understand as it could have important mental health implications. Research to date has generally suggested negative mental health impacts of home confinement. For instance, higher levels of psychological distress (Ammar et al., 2020b; Niedzwiedz et al., 2021) and loneliness (Bu, Steptoe, & Fancourt, 2020) were found during the COVID-19 lockdown compared to before. It was also found that depressive and anxiety symptoms peaked during lockdown which decreased following the easing of restrictions (Fancourt et al., 2021). Potential mechanisms include but not limited to the feeling of infringement upon personal freedom, limited access to health care, reduced social participation and support, related financial loss or other adversities (Ammar et al., 2020a; Pfefferbaum & North, 2020). It is important to note that most of the existing evidence came from studies comparing lockdowns to non-lockdown periods. However, although lockdown limited how much people could leave their homes, it did allow people to go outside each day, which is not the same as home confinement. There has been little research on the mental health implications of home confinement more specifically.

Further, there has been limited research so far into who demographically were more likely to engage in home confinement during the COVID-19 pandemic. Recent evidence on ‘compliance’ showed that younger age, male gender, higher educational level and living alone were associated with lower levels of compliance (Wright & Fancourt, 2021). Another study found that age, gender, education, employment status, living alone, physical and mental health, personality were related to the growth trajectory of compliance (Wright, Steptoe, & Fancourt, 2021). However, there is a substantial distinction between ‘compliance’ and ‘home confinement’. First, the COVID-19 rules and guidelines were broad and included hand hygiene, face covering, social distancing and so forth, so using ‘compliance’ as a proxy for understanding home confinement is flawed. Moreover, as going outdoors was not prohibited even during strict lockdown periods in England as long as it was for essential reasons such as exercise, home confinement might in fact be only weakly related to compliance. Indeed, leaving one's home for exercise was still recommended for maintaining good health, so one could still fully comply with the lockdown ‘stay-at-home’ orders but nonetheless leave the home every day. Given the possible negative mental health effects of home confinement, it is important to identify who engaged in this behaviour so that resources can be targeted to support these individuals if needed.

Therefore, this study examined home confinement defined as staying at home without leaving the property. We used data from 25 390 adults living in England who were followed for 17 months between March 2020 and July 2021. Data were analysed using the growth mixture modelling (GMM) approach allowing for different patterns of longitudinal changes in home confinement. Further, this study sought to explore factors associated with longitudinal patterns of home confinement and their mental health implications. This will facilitate a better understanding of behavioural changes during COVID-19 and shed light on policy and interventions.

## Methods

### Study design and participants

This study analysed data from the University College London (UCL) COVID-19 Social Study, a large panel study of the psychological and social experiences of over 75 000 adults (aged 18+) in the UK during the COVID-19 pandemic. The study commenced on 21 March 2020 and involved weekly and then monthly (4-weekly) online data collection from participants during the pandemic. The study did not use a random sample design and therefore the original sample is not representative of the UK population. However, it does contain a heterogeneous sample that was recruited using three primary approaches. First, convenience sampling was used, including promoting the study through existing networks and mailing lists (including large databases of adults who had previously consented to be involved in health research across the UK), print and digital media coverage, and social media. Second, more targeted recruitment was undertaken focusing on (i) individuals from a low-income background, (ii) individuals with no or few educational qualifications, and (iii) individuals who were unemployed. Third, the study was promoted via partnerships with third sector organisations to vulnerable groups, including adults with pre-existing mental health conditions, older adults, carers, and people experiencing domestic violence or abuse. The study was approved by the UCL Research Ethics Committee (12467/005) and all participants gave informed consent. Detailed information on the study is available online at https://osf.io/jm8ra/.

In this study, we restricted the sample to participants living in England (*N* =58 486). Further, we included only participants with at least three repeated measures between 21 March 2020 and 25 July 2021. These criteria provided us with data from 37 800 participants. Around 10% of these participants had missing data for demographic, social and health-related predictors and another 3.1% for mental health measures. After excluded these participants, we had a sample size of 32 856 participants. Finally, we excluded keyworkers (people working in essential sectors such as health and social care, transport etc.) who were in employment, given they were likely to have an established pattern of home confinement especially during lockdowns. This provided us with a final analytical sample of 25 390 participants who were followed up for a maximum of 17 months.

## Measures

### Home confinement

Participants were asked every week: ‘In the past 7 days, how many days have you not left the house or garden?’ The options ranged from 0 to 7 days. This was analysed as a continuous variable.

### Predictors

We considered a range of socio-demographic, social, health and psychological factors as potential predictors. These included gender (women *v.* men), ethnicity (white *v.* ethnic minorities), age groups (age 18–29, 30–45, 46–59, 60+), education (up to GCSE levels, A-levels or equivalent, and university degree or above), income (<£30 000 *v.* ⩾£30 000 per annum), employment status (employed *v.* other), area of living (rural *v.* urban), dwelling type (house *v.* other), crowded household defined as average room (excluding bathroom/toilet) per person ⩽1 (yes *v.* no) and dog ownership (yes *v.* no). Social factors included living situation (living alone *v.* living with others), number of close friends (0 to 10+) and usual social contacts (1 to 5, from less than once a month to every day). In addition, we included two health-related factors: self-reported diagnosis of any long-term physical health condition (e.g. asthma or diabetes) or any disability (yes *v.* no), and self-reported diagnosis of any long-term mental health condition (e.g. depression, anxiety) (yes *v.* no). Psychological factors included personality traits (neuroticism, extraversion, openness, agreeableness and conscientiousness) and COVID-19 stress. The former was measured by the Big Five Inventory (BFI-2) (Soto & John, 2017). The latter was measured by asking participants if they had experienced any minor/major stress about catching COVID-19 or becoming serious ill from it. This was coded as none, minor stress and major stress.

### Distal outcomes

To explore the mental health implications of home confinement, we looked at depressive and anxiety symptoms. Depressive symptoms were measured using the Patient Health Questionnaire (PHQ-9) (Löwe, Kroenke, Herzog, & Gräfe, 2004), a standardised instrument for screening for depression in primary care. Unlike the original PHQ-9, the current study enquired about symptoms ‘over the last week’ instead of ‘over the last two weeks’ as data were initially collected weekly. The questionnaire includes nine items with 4-point responses ranging from ‘not at all’ to ‘nearly every day’. Higher overall scores indicate more depressive symptoms, ranging from 0 to 27. Anxiety symptoms were measured using the Generalized Anxiety Disorder Assessment (GAD-7) (Spitzer, Kroenke, Williams, & Löwe, 2006), a well-validated tool used to screen for generalised anxiety disorder in clinical practice and research. These questions were also worded as ‘over the last week’ for the same reason as the depression items. The GAD-7 comprises seven items with 4-point responses ranging from ‘not at all’ to ‘nearly every day’, with higher overall scores indicating more symptoms of anxiety, ranging from 0 to 21. Both depressive and anxiety symptoms were measured longitudinally. We used measures at the last time point when participants were observed as distal outcomes, while controlling for the relevant mental health measures at the baseline.

### Statistical analysis

Data were analysed using GMM. The conventional growth modelling approach assumes one homogeneous growth trajectory, allowing individual growth factors to vary randomly around the overall mean. GMM relaxes this assumption and enables researchers to explore different patterns of change (latent trajectory classes) (Muthén & Asparouhov, 2008). More specifically, we used GMM with free time scores, where time scores (months) were estimated as free parameters, except for two fixed at 0 and 1 for the purpose of model identification. In this model, we made no assumption about the shape of growth trajectories which was left to be determined by data.

Starting with the unconditional GMM (Model I), we compared models with a different number of classes on the basis of the Bayesian information criterion (BIC) and sample-size adjusted Bayesian information criterion (ABIC), along with the Vuong-Lo-Mendell-Rubin likelihood ratio (LMR-LR) test and Adjusted Lo-Mendell-Rubin likelihood ratio (ALMR-LR) test. After identifying the optimal number of classes, we introduced covariates to the model to explore what factors were associated with class membership, using the logistic regression model (Model II). Next, we included mental health measures as distal outcomes (Model III), with depressive and anxiety symptoms being modelled separately in linear regression controlling for baseline depression/anxiety in addition to Model II covariates (see Figure S2 in the Supplement). This allowed us to examine how latent classes (LCs) were related to mental health at the end of the follow-up. These models were fitted using the new three-step approach: (1) to conduct unconditional GMM; (2) to estimate the most likely class membership from the posterior probabilities and measurement errors; (3) to estimate the relationship of the most likely class membership with covariates or distal outcomes, correcting for measurement errors in the estimation of LC membership (Vermunt, 2010). Weights were applied throughout the analyses. The analytical sample (before excluding keyworkers) was weighted to the proportions of gender, age, ethnicity and education in the English population obtained from the Office for National Statistics (Office for National Statistics, 2019). The main analyses were implemented in Mplus V8.

## Results

### Sample characteristics

Before weighting the 25 390 participants, there was an over-representation of women and people with a degree or above and an under-representation of people from ethnic minority backgrounds and adults under 30 (Table 1). After weighting, the sample reflected population proportions, with 50.7% women, 34.6% with a degree or above, 12.3% of ethnic minority and 17.9% under 30.

**Table 1.** Descriptive statistics of the sample before and after weighting (*N* = 25 390)

	Raw data		Weighted data	
	%/Mean (s.d.)	*N*	%/Mean (s.d.)	*N*
Gender				
Women	74.1	18 807	50.7	12 714
Men	25.9	6583	49.3	12 342
Ethnicity				
Minority	4.5	1152	12.3	3090
White	95.5	24 238	87.7	21 966
18–29	6.3	1607	17.9	4486
Age				
30–45	24.6	6250	24.2	6064
46–59	30.3	7699	22.0	5516
60+	38.7	9834	35.9	8990
Education				
Low (GCSEs or below)	13.6	3463	33.6	8424
Medium (A-levels or equivalent)	16.8	4275	31.8	7966
High (Degree or above)	69.5	17 652	34.6	8666
Household income (annual)				
<30k	40.4	10 269	49.3	12 342
⩾30k	59.6	15 121	50.7	12 714
Employment status				
Employed	54.5	13 833	48.5	12 148
Other	45.5	11 557	51.5	12 908
Area of living				
Urban	78.2	19 865	79.7	19 980
Rural	21.8	5525	20.3	5076
Dwelling type				
House	78.8	19 995	74.5	18 687
Other	21.2	5395	25.4	6369
Overcrowded				
Yes	9.4	2389	15.3	3683
Household				
No	90.6	23 001	84.7	21 373
Dog ownership				
Yes	22.3	5651	23.4	5863
No	77.7	19 739	76.6	19 193
Living status				
Living alone	21.7	5514	19.8	4961
Living with others	78.3	19 876	80.2	20 095
Number of close friends (range: 0–10)	4.9 (3.1)	25 390	4.5 (3.1)	25 056
Usual social frequency (range: 1–5)	3.1 (1.1)	25 390	3.0 (1.2)	25 056
Physical health condition				
Yes	39.4	9996	39.9	9995
No	60.6	15 394	60.1	15 061
Mental health condition				
Yes	17.8	4518	20.6	5150
No	82.2	20 872	79.4	19 906
Big Five personality (range: 3–21)				
Neuroticism	11.3 (4.3)	25 390	11.5 (4.5)	25 056
Extraversion	12.9 (4.3)	25 390	12.6 (4.3)	25 056
Openness	15.5 (3.3)	25 390	15.0 (3.3)	25 056
Agreeableness	15.5 (3.1)	25 390	15.3 (3.2)	25 056
Conscientiousness	15.8 (3.0)	25 390	15.5 (3.1)	25 056
COVID-19 stress				
None	40.0	10 155	42.5	10 648
Minor stress	37.2	9452	34.0	8528
Major stress	22.8	5783	23.5	5880
Depressive symptoms baseline (range: 0–27)	6.4 (5.8)	25 390	7.0 (6.3)	25 056
Depressive symptoms last wave (range: 0–27)	5.6 (5.8)	25 390	6.1 (6.3)	25 056
Anxiety symptoms baseline (range: 0–21)	5.3 (5.2)	25 390	5.6 (5.6)	25 056
Anxiety symptoms last wave (range: 0–21)	4.2 (5.1)	25 390	4.6 (5.5)	25 056

### Latent trajectory classes

**Fig. 1.**
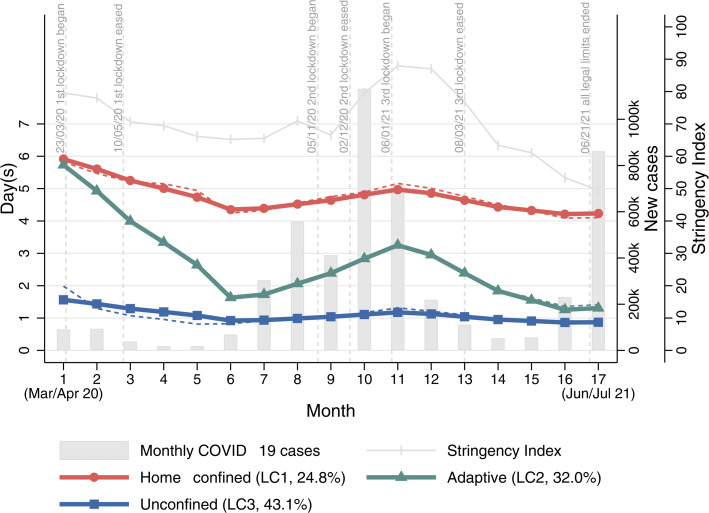
Estimated growth trajectories of home confinement for each latent class from the unconditional GMM model (Model I).

The three-class GMM model was chosen as the optimal model based on likelihood ratio tests (Online Supplementary Table S2). It had an adequate quality of class membership classification (entropy = 0.66). Figure 1 shows the estimated growth trajectory of home confinement for each LC. The figure also includes the stringency index of the strictness of government policies in England, with higher scores indicating greater strictness (Hale et al., 2021), and the number of new cases of COVID-19 (Government of the United Kingdom, 2021a).

The first class (24.8%) was marked by a high level of home confinement (4–6 days a week) persisting throughout the 17-month period, even when lockdown measures were eased with the stringency index being relatively low. Therefore, it was labelled as the ‘home-confined’. However, this class did show a decline in days in confinement between March and August 2020 before it started to increase in September 2020 and then decrease again around February 2021. This trend was in line with the changes in national policies and to a lesser extent with the number of COVID-19 cases with numbers being relatively low at the start (Fig. 1). The second class (32.0%) started off with a high level of confinement at the beginning of the first national lockdown, which declined sharply (from 6 to 1.7 days a week) into summer months. This was followed by an increase from September 2020 and peaked in January 2021 when the third national lockdown started, before it started to decrease again. This class was labelled as the ‘adaptive’ who seemed to adapt their behaviours to circumstance changes to a much greater extent compared to the ‘home-confined’. The third class was the largest (43.1%), which consisted of people showing a very low level of home confinement consistently (about 1 day a week) regardless of policy changes and COVID-19 cases. They were regarded as the ‘unconfined’.

### Factors associated with latent trajectory classes

We fitted a logistic regression model to examine how individual characteristics were related to class membership of home confinement trajectories, using LC1 (‘home-confined’) as the reference (Table 2). Women had 24% lower odds of being adaptive (LC2) and 42% lower odds of being unconfined (LC3) than men. There was no ethnic difference comparing LC2 to LC1, but people from ethnic minority backgrounds had 33% lower odds of being unconfined (LC3). People aged 46 or above had lower odds of being adaptive [odds ratio (OR) = 0.55], but older adults aged 60 or above had 50% high odds of being in the unconfined class than young adults aged 18–29. There was no educational difference comparing LC2 to LC1, but those with a higher education qualification had 57% higher odds of being unconfined. People from low-income households had 24% lower odds of being adaptive and 36% lower odds of being unconfined. People who were employed at the baseline had 2.4 times odds of being adaptive and 2.41 times odds of being unconfined than those who were not in employment. There was no evidence that rurality or dwelling type was related to latent trajectory classes. People living in the overcrowded household had 26% lower odds of being unconfined. There was no evidence that dog ownership was related to the odds of being adaptive, but people who owned a dog had 2.69 times odds of being unconfined.

**Table 2.** Predictors of the latent class membership from Model II (*N* = 25 390)

	Adaptive (v. Home-confined) LC2 (*v.* LC1)		Unconfined (*v.* Home-confined) LC3 (*v.* LC1)	
	OR	95% CI	OR	95% CI
Women (*v.* men)	**0.76**	**(0.62–0.94)**	**0**.**58**	**(0.49–0.68)**
Ethnic minority (*v.* white)	1.02	(0.70–1.50)	**0**.**67**	**(0.47–0.95)**
Age: 30–45 (*v.* 18–29)	0.91	(0.63–1.31)	1.21	(0.87–1.69)
Age: 46–59 (*v.* 18–29)	**0**.**55**	**(0.38–0.79)**	1.26	(0.91–1.74)
Age: 60+ (*v.* 18–29)	**0**.**55**	**(0.38–0.81)**	**1**.**50**	**(1.07–2.09)**
Education: A levels (*v.* GCSEs or below)	1.01	(0.79–1.28)	1.15	(0.95–1.40)
Education: degree + (*v.* GCSEs or below)	0.89	(0.69–1.13)	**1**.**57**	**(1.30–1.89)**
Low income: <30k (*v.* ⩾30k)	**0**.**76**	**(0.61–0.94)**	**0**.**64**	**(0.54–0.76)**
Employed (*v.* other)	**2**.**40**	**(1.93–2.99)**	**2**.**41**	**(2.02–2.89)**
Rural (*v.* urban)	1.16	(0.92–1.46)	0.92	(0.77–1.11)
Living in a house (*v.* other)	1.05	(0.82–1.35)	0.85	(0.70–1.03)
Overcrowded (*v.* no)	1.01	(0.74–1.39)	**0**.**74**	**(0.56–0.97)**
Own a dog (*v.* none)	1.02	(0.81–1.30)	**2**.**69**	**(2.24–3.23)**
Living alone (*v.* with others)	**0**.**70**	**(0.54–0.91)**	0.91	(0.75–1.10)
Number of close friends	**1**.**05**	**(1.01–1.09)**	**1**.**06**	**(1.03–1.09)**
Frequency of social contacts	**1**.**35**	**(1.23–1.47)**	**1**.**43**	**(1.33–1.54)**
Physical health diagnosis (*v.* no)	**0**.**65**	**(0.53–0.79)**	**0**.**36**	**(0.30–0.42)**
Mental health diagnosis (*v.* no)	**0**.**69**	**(0.54–0.89)**	**0**.**60**	**(0.49–0.74)**
Personality: neuroticism	1.02	(1.00–1.05)	1.02	(1.00–1.04)
Personality: extraversion	**1**.**04**	**(1.02–1.07)**	**1**.**03**	**(1.01–1.05)**
Personality: openness	0.98	(0.95–1.01)	**0**.**96**	**(0.94–0.99)**
Personality: agreeableness	0.99	(0.96–1.02)	**0**.**96**	**(0.94–0.99)**
Personality: conscientiousness	1.02	(0.99–1.06)	**1**.**08**	**(1.05–1.11)**
COVID-19 stress minor (*v.* none)	1.15	(0.92–1.43)	0.92	(0.77–1.09)
COVID-19 stress major (*v.* none)	0.88	(0.69–1.12)	**0**.**58**	**(0.47–0.70)**

All social factors were found to be associated with latent trajectory classes. People living alone had 30% lower odds of being adaptive, but no difference was found comparing LC3 to LC1. People with more close friends had higher odds of being both adaptive [OR 1.05, 95% confidence interval (CI) 1.01–1.09] and unconfined (OR 1.06, 95% CI 1.03–1.09). Similarly, frequent social contacts were also associated with higher odds of being adaptive (OR 1.35, 95% CI 1.23–1.47) and unconfined (OR 1.43, 95% CI 1.33–1.54).

People with pre-existing physical health conditions had 35% lower odds of being in the adaptive class and 64% lower odds in the unconfined class. Similarly, people with mental health conditions had 31% lower odds of being adaptive and 40% lower odds of being unconfined. A personality trait, extraversion was associated with higher odds of being adaptive (OR 1.04, 95% CI 1.02–1.07). Extraversion and conscientiousness were also associated with higher odds of being unconfined (OR 1.03–1.08); whereas openness and agreeableness were associated with lower odds of being unconfined (OR 0.96). Finally, there was little evidence that stress was related to latent trajectory classes, except that having major stress related to COVID-19 at baseline were associated with lower odds of being unconfined (OR 0.58, 95% CI 0.47–0.70).

### Mental health by latent trajectory classes

**Fig. 2.**
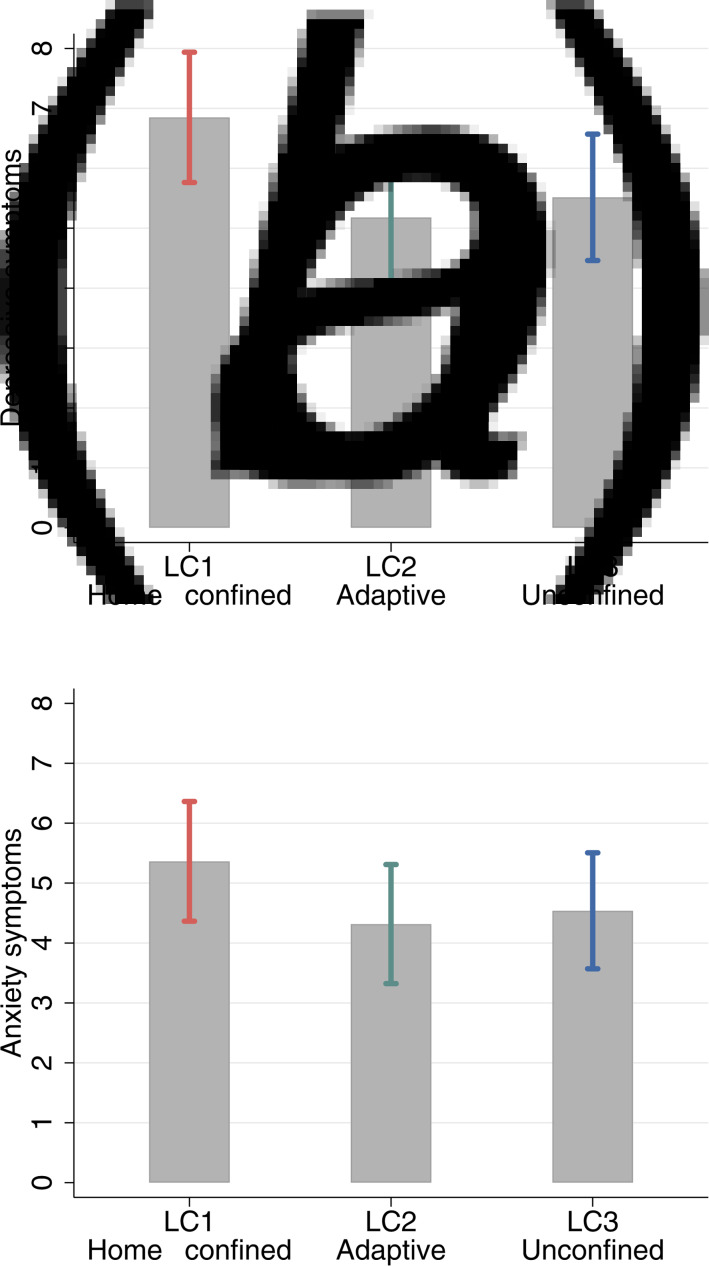
Estimated depressive and anxiety symptoms at the end of the follow-up by class membership from Model III (Notes: Categorical predictors were set to use the reference and continuous variables to the mean, additionally controlling for depressive/anxiety measure at baseline respectively.).

Models including mental health measures as distal outcomes addressed the question whether the patterns of home confinement trajectories were related to depressive and anxiety symptoms. The estimated depressive and anxiety symptoms and their 95% confidence intervals by LCs are presented in Fig. 2. People in the home-confined class had the highest number of depressive symptoms independent of all covariates and depressive symptoms at baseline, followed by the unconfined. The adaptive class had the lowest number of depressive symptoms. All class differences were statistically significant (LC1-LC2: diff = 1.67, *p* < 0.001; LC1-LC3: diff = 1.33, *p* < 0.001; LC3-LC2: diff = 0.34, *p* = 0.028). Similar results were also found for anxiety symptoms. The home-confined had higher levels of anxiety than the adaptive (diff = 1.05, *p* < 0.001) and unconfined (diff = 0.83, *p* < 0.001). However, the difference between the adaptive and unconfined was not statistically significant (diff = 0.22, *p* = 0.135).

## Discussion

This study is the first to examine the longitudinal changes in home confinement during the COVID-19 pandemic. Using data from England, our analyses identified three unique classes of home-confinement trajectories. About one in four participants in the weighted sample had a high level of confinement persistently across the 17-month follow-up (March 2020-July 2021). Nearly a third participants started with a high level of confinement which decreased sharply into the summer months, then rose and fell in accordance to changes in government policies. The largest proportion of participants (43%) had little change over time, showing a very low level of home confinement.

The class membership was related to a range of factors. Most likely to be in the home-confined class were those with pre-existing physical health conditions. This was as expected given guidance from the World Health Organization and National Health Service advising people with specific health conditions to shield. People who were shielding were asked not to go outdoors at all during the first few weeks of the pandemic, although as the first lockdown eased, they were permitted to go outdoors to exercise. Nonetheless, caution may have limited their willingness to do so. Notably, these people were reported having poorer psychosocial experience during the pandemic (Bu et al., 2020; Fancourt et al., 2021), which might be partially explained by their high level of home confinement. People with pre-existing mental health conditions were also more likely to engage in persistent home confinement, but interestingly neither neuroticism nor being stressed about catching or becoming ill from COVID-19 was related to engaging in home confinement rather than simply following the guidelines (‘adaptive’ class). This suggests two things. First, concern about the virus itself did not lead people to impose stricter rules on their own behaviours than the national guidance (although people who were majorly stressed about COVID-19 were less likely to be in the ‘unconfined’ class). Second, pandemic-related stress itself or general neurotic traits did not lead people to confine themselves to their homes, but rather pre-existing mental illness was the specific predictor.

Amongst other predictors, women were more likely to engage in home confinement than men. Recent studies generally reported a higher level of psychological distress among women during COVID-19 (Fancourt et al., 2021; Xue & McMunn, 2021), so it is possible that the higher level of home confinement amongst women partly explain these prior findings. In considering why, our findings were independent of other factors that could plausibly explain this relationship such as stress and mental health (both higher in women during the pandemic). But one explanation could lie in the division of labour: women were found to spend more time on unpaid care and more likely to reduce working hours to accommodate increasing childcare demand during lockdowns (Xue & McMunn, 2021). This may have reduced available time to go outdoors for fresh air or exercise. Our findings for age were complex. Adults aged 60+ were more likely to be the classes of home-confined and unconfined. This suggests a heterogeneity in older people's responses to COVID-19, and highlights that there was more nuance in older adults' behaviours than merely the higher compliance among older people often reported (Wright & Fancourt, 2021; Wright et al., 2021). Notably, the two classes that were associated with older age showed relatively little change over time. With increasing awareness of the importance of maintaining mental wellbeing during COVID-19 (Smith et al., 2021), it is possible that older adults were keen to stick to their existing routines, using going out as a means to be physically active and to cope with mental health challenges. As reported in a qualitative study, some older adults focused more on their health during COVID-19 and became physically more active by going for regular walks and taking up new forms of physical activity (McKinlay, Fancourt, & Burton, 2021).

Another factor that was associated with higher odds of being home-confined persistently is low household income. As we only controlled for employment status at the baseline, it is probable that people from low-income households were likely to be in jobs that were more subject to unemployment and furlough schemes during the follow-up (Botha, de New, de New, Ribar, & Salamanca, 2020; Carta & de Philippis, 2021), which could contribute to why they were less likely to have daily reasons to leave the house for work. But it does not fully explain why their home confinement would be persistently higher.

Social factors were also found to predict class membership. Adults who lived alone were more likely to engage in home confinement (relative to being adaptive). The small size of the friend network and less frequent social contacts were also associated with higher odds of persistently engaging in home confinement. These findings suggest that people who were socially more isolated at the start of the pandemic were at risk of a high level of home confinement throughout the pandemic, which in turn might have exacerbated their sense of social isolation. These individuals were not merely less extrovert, as although adults who scored lower on extraversion were consistently more likely to be home-confined, our analyses simultaneously controlled for extraversion, so existing social isolation remained an independent risk factor. Instead, the mechanism may be related to a lack of social support and motivation, and unhealthy lifestyles, such as lower physical activity and more sedentary behaviours as suggested in previous literature (Leigh-Hunt et al., 2017). This might be exacerbated during the COVID-19 pandemic when usual social and daily routines were disrupted.

Furthermore, this study addressed another important question about whether patterns of home-confinement trajectories were related to mental health outcomes. Our analyses revealed a much higher number of depressive and anxiety symptoms among people who engaged in home confinement persistently. This is in line with well-established evidence of the negative mental health impact of social isolation in general prior to COVID-19 (Cacioppo & Hawkley, 2003; Courtin & Knapp, 2017), and recent evidence linking lockdowns with a range of negative outcomes such as loneliness, life satisfaction, depressive symptoms (Ammar et al., 2020a, 2020b; Bu et al., 2020). However, it is worthy of note that people in the unconfined class had more depressive symptoms than those who were adaptive. Similar finding was also found for anxiety symptoms albeit statistically insignificant. One possible explanation is that for some going out of the house was not necessarily by choice but for practical reasons, for example work or other obligations such as caring (even though we excluded keyworkers). In other words, it is possible that some people in the unconfined class were unable rather than unwilling to reduce their days of going out, especially during national lockdowns. Although going outdoors comes with mental health benefits in general, this aspect of external obligations and the associated health risks may have had a negative impact on people's mental health, which may explain the difference in depressive symptoms between the unconfined and adaptive.

This study has a number of strengths including its large sample size, repeated monthly follow-up of the same participants over 17 months since the first UK lockdown across three national lockdown periods, and robust statistical approaches. Although the UCL COVID-19 Social Study did not use a random sample, it does have a large sample size with wide heterogeneity, including good stratification across all major socio-demographic groups. In addition, analyses were weighted on the basis of population estimates of core demographics, with the weighted data showing good alignment with national population statistics and another large scale nationally representative social survey (Bu et al., 2020). Despite all efforts to make our sample inclusive (e.g. by targeted recruitment) and representative (by weighting), we cannot rule out the possibility of selection bias due to factors associated with survey participation which are not accounted for by weighting (e.g. internet access, topic interest). We therefore advise caution when generalising these findings to the population. Further, there is a lack of pre-pandemic information. Therefore, it is unclear how the growth trajectories of home confinement are related to patterns before the pandemic, and it is possible that some of the individuals in the home-confined category habitually spend more of their time at home.

Lockdowns and stay-at-home orders have been shown to be essential and effective in controlling the COVID-19 outbreak, but there have been concerns about their potential negative impacts on the mental health of the public (Brooks et al., 2020; Pfefferbaum & North, 2020). Our study showed that most adults in the weighted sample had a persistently low level of home confinement or adjusted their confinement level in according to policy changes. However, one in four adults in the weighed sample maintained a high level of home confinement throughout, even during periods when containment measures were eased or removed and when infection rates were low. These findings are concerning as our analyses have shown that persistent home confinement is related to worse mental health outcomes. It is promising that the mental health impact of COVID-19 is well acknowledged and the UK Government has set out an action plan during 2021 to 2022 to address these issues (Government of the United Kingdom, 2021b). Based on findings from this study, we advocate that persistent home confinement should be given special attention in future action plans and interventions. In addition to services for the general public, targeted interventions for at-risk groups (e.g. women, people with pre-existing physical or mental health conditions) are also needed.

## Data Availability

Anonymous data will be made publicly available following the end of the pandemic.

## Supplementary material

For supplementary material accompanying this paper visit https://doi.org/10.1017/S0033291722000605. click here to view supplementary material
